# Notes on Preparations for the Sick

**Published:** 1893-09

**Authors:** 


### Notes on Preparations for the Sick.

---

**Kumysgen. Lacto-cereal Food. Lacto-Preparata. Pancrobilin. Pills: Pancrobilin; Pancrobilin Comp.**—Reed and Carnrick, New York.—Through Messrs. Henry Hodder and Co., the sole agents for Bristol, we have received samples of these preparations, which are still comparatively new and not much employed in this country. They have much in their favour, and should become as popular as their useful predecessors — the Beef Peptonoids, the Soluble Food, the Cod-Liver Oil Milk, and other preparations of the same firm.

**Kumyss** has of late fallen into comparative disuse, peptonised milk having supplanted it. The older preparations of kumyss were so difficult to obtain in emergencies that they were very naturally discarded for something more attainable, and we had begun to think that kumyss was quite a thing of the past. The improved method of preparation by means of powder or tablet —uniform, permanent, and always ready for use—has again given it a chance of competing successfully with other kinds of predigested milk. By means of kumysgen it is possible to prepare excellent kumyss with rapidity and ease, and the result must sometimes be more satisfactory than that given by any process of peptonisation. In cases of continued vomiting it is likely to be of the greatest service, and more especially to the numerous patients who dislike milk, no matter how well it may be peptonised, or for whom the effervescent preparation is especially applicable.

**Lacto-cereal Food** is composed of powdered milk, sterilised and partly digested (50 per cent.); dextrinated cereals, desiccated bananas, cocoa butter, and manna. It should prove to be a highly nutritious, easily digested, and restorative food in all cases of defective nutrition.

**Lacto-Preparata** is a purely all-milk food for nursing infants. It is a fine powder, free from starch, and carefully sterilised.

The **Pancrobilin** preparations are intended to relieve all varieties of intestinal and hepatic derangements — a large undertaking.

---

**Pancreatic Emulsion.**—Savory and Moore, London.—The many recently introduced emulsions and so-called solutions of fats are a little apt to supplant an old and much-valued one which has done good service for many years. A reminder is therefore necessary that it has not deserted us. We question if any better or more useful preparation as a substitute for cod-liver oil can yet be said to exist. It has usually given excellent results when we have had occasion to use it.

---

**Duncan's Compound Syrup of the Hypophosphites. Liq. Santal. Flav. c̄ Cub. et Buchu. Syr. Ferri. et Quin. Hydrobrom. c̄ Strych. Liq. Papain et Iridin. Co. Pancreatinum Fluidum. Capsules: Compound Iron; Blaud's Pill; Cascara.**—DUNCAN, FLOCKHART AND CO., Edinburgh.—These preparations must be well known to all who legitimately use drugs, and the reputation of the firm is a guarantee of their good quality.

---

**Palma Christi (Dr. Standke).**—T. CHRISTY & CO., London.—This name is given to a pure and active preparation of castor oil. It is pleasantly flavoured with aromatics, and in appearance is not unlike an aromatic syrup.

---

**Acid Glycerine of Pepsine.**—T. BUXTON, Clifton.—We have here an excellent preparation of pure pepsine in an agreeable form. One to two teaspoonfuls after meals give much relief in cases of atonic dyspepsia.

---

**Abdomen Bandage.**—S. MAW, SON AND THOMPSON, London.—Messrs. Maw, Son and Thompson have sent us a specimen of their silk elastic abdomen bandages, so constructed as to admit of its being expanded or contracted under varying circumstances. It is simple as well as effectual, and can be easily regulated by the patient. It is made light and cool so as to be easily borne, and is especially well adapted for cases of pregnancy, but can be used also in cases of ascites or ovarian disease.

---

**Dermatol Dusting Powder. Elite Toilet Lanoline Cream.**—BURROUGHS, WELLCOME AND CO., London.—The Powder is a diluted preparation of Dermatol, suitable for dusting over irritable conditions of the skin, such as blisters or abrasions. It is very smooth and soothing, possessing slightly astringent as well as antiseptic properties. It is free from all offensive smell, and can be used for children and general nursery purposes. We have tried the specimen sent to us, and can speak of it with warm approval.

The Cream is an elegantly-mounted preparation, combining novelty and efficiency as a soothing and antiseptic application for protecting and preserving the skin.

---

**Golden Hop Ale.**—J. & T. Usher, Bristol.—This is a comparatively new candidate for public favour, belonging to that class of beverage called (for want of a better name) "non-intoxicating." It is an exceedingly clean and apparently wholesome drink. During the recent hot weather we found it very pleasant and exhilarating. We have no hesitation in recommending it as one of the best of its kind. It is supplied in cask and in bottle. Perhaps its aromatic flavour is a trifle too pronounced, but that could be easily remedied. It is not at all unlike the more alcoholic "bitter" which it is intended to rival.

---

In March, 1892, we noticed with favour some calf lymph sent us by Mr. Rebman from the Institute of Dr. Pissin of Berlin. We have just received some more tubes of this lymph, for the sale of which Mr. Buxton of Queen's Road has been appointed the sole agent for this neighbourhood.

